# Atopic Dermatitis and the Risk of Infection in End-Stage Renal Disease

**DOI:** 10.3390/medicina59122145

**Published:** 2023-12-10

**Authors:** Rushan I. Momin, Stephanie L. Baer, Jennifer L. Waller, Lufei Young, Sarah Tran, Varsha Taskar, Wendy B. Bollag

**Affiliations:** 1 Medical College of Georgia, Augusta University, Augusta, GA 30912, USA; rmomin@augusta.edu (R.I.M.); stephanie.baer@va.gov (S.L.B.); jwaller@augusta.edu (J.L.W.); sarahytran@gmail.com (S.T.); vtaskar@augusta.edu (V.T.); 2 Charlie Norwood Department, Veterans Affairs Medical Center, Augusta, GA 30904, USA; 3 School of Nursing at UNC Charlotte, University of North Carolina Charlotte, Charlotte, NC 28223, USA

**Keywords:** atopic dermatitis, end-stage renal disease, infection, bacteremia, septicemia, cellulitis, herpes zoster, conjunctivitis

## Abstract

*Background and Objectives*: Atopic dermatitis (AD), also known as eczema, is a common chronic inflammatory skin condition affecting 16.5 million adults in the United States. AD is characterized by an impaired epidermal barrier that can predispose individuals to infection. End-stage renal disease (ESRD) is also commonly complicated by infections due to chronic vascular access and immune-system dysfunction, possibly related to uremia. Multiple studies have reported that renal disease is a common comorbidity in adults with atopic dermatitis. The aim of this study was to determine whether AD is a risk factor for certain infections in patients with ESRD. *Materials and Methods*: Using the United States Renal Data System, a retrospective cohort analysis was conducted on adult ESRD patients initiating dialysis between 2004 and 2019 to investigate associations between infections and AD in this population. *Results*: Of 1,526,266 patients, 2290 were identified with AD (0.2%). Infectious outcomes of interest were bacteremia, septicemia, cellulitis, herpes zoster, and conjunctivitis. In all infectious outcomes except for conjunctivitis, patients with the infectious outcomes were more likely to carry a diagnosis of AD. After controlling for demographic and clinical covariates, AD was associated with an increased risk of cellulitis (adjusted relative risk (aRR) = 1.39, 95% confidence interval (CI) = 1.31–1.47) and herpes zoster (aRR = 1.67, CI = 1.44–1.94), but not with bacteremia (aRR = 0.96, CI = 0.89–1.05), septicemia (aRR = 1.02, CI = 0.98–1.08), or conjunctivitis (aRR = 0.97, CI = 0.740–1.34). *Conclusions*: Overall, after controlling for demographic and clinical covariates and adjusting for person-years-at-risk, AD was associated with an increased risk for some, but not all, infections within the population of patients with ESRD.

## 1. Introduction

Atopic dermatitis (AD), also known as eczema, is a common chronic inflammatory skin condition impacting 31.6 million people in the United States. The prevalence of AD has reached epidemic magnitude, especially in urban populations [1]. This rapid increase is attributed to multiple factors, including environmental, lifestyle, and genetic predisposing factors [1]. Environmental factors include drastic changes in seasonality and climate, increased exposure to traffic-induced air pollution and smoking, and increased water hardness [1]. Increased psychological stress is also associated with the rising prevalence of AD. Lifestyle factors, such as skincare practices, are another major influence on AD prevalence. The frequent use of soaps and detergents contributes to skin-barrier dysfunction, promotes skin inflammation, and leads to the development of AD. AD is commonly associated with children, affecting up to 20% of children; however, AD also impacts up to 10% of adults, ranking 15th worldwide for non-fatal diseases and first for skin diseases, measured in disability-adjusted life years [2,3,4].

AD is characterized by an impaired epidermal skin barrier with symptoms including pruritis, erythema, and xerosis [2,5]. Adult-onset AD exhibits phenotypical differences compared with childhood-onset AD, such as a decreased association with family history, higher probability of lesions on the head and hands, and lower probability of flexural lesions [6]. In addition, children with the early-onset AD often “outgrow” the disease, with apparent correction of the skin lesions [7]. Nevertheless, there is some confusion as to whether childhood- and adult-onset AD are the same or distinct entities [7].

The pathogenesis of AD involves T cells, elevated IgE, and cytokines IL-4, IL-5, and IL-13 [5,8,9]. Some of the genes linked to the process of AD include those encoding proteins involved in the differentiation of keratinocytes and the maintenance of the skin barrier [10]. With an impaired epidermal barrier, there is dysregulation of the skin microbiome as seen by the increased *Staphylococcus aureus* colonization [11]. The combination of skin-barrier defects, an overactive immune system, and skin-microbiome dysfunction allows for an increased risk of viral and bacterial infections [12].

End-stage renal disease (ESRD) is a condition in which the kidneys cease functioning, such that patients with ESRD require long-term dialysis or a transplant to survive. Many patients live with the implications of ESRD, which include premature mortality and reduced quality of life [13]. The immune system of ESRD patients is impacted primarily due to chronic uremia. There may be increased proinflammatory cytokines due to decreased renal function or uremia-induced generation of cytokines [14]. Furthermore, uremia is also associated with immunosuppression by decreasing the function of monocytes, neutrophils, and dendritic cells [14]. Given the state of chronic uremia, as well as continuing vascular access for dialysis, patients with ESRD are prone to many infectious complications that contribute to morbidity and mortality [15,16].

Previous studies reporting the connection between AD and chronic kidney disease are conflicting. A recent 2023 study suggested a bidirectional positive association between AD and chronic kidney disease such that AD was associated with an increased risk of chronic kidney disease, and chronic kidney disease was associated with an increased risk of AD [17]. A case-control study found chronic kidney disease to be weakly associated with AD, as well as with other skin conditions such as psoriasis and hidradenitis suppurativa, such that people with stages 3–5 chronic kidney disease were more likely than controls to have these skin conditions [18]. However, in the cohort with diabetes mellitus, there was no positive association between AD and chronic kidney disease [18]. Factors underlying these associations may be electrolyte imbalances, increased uremic substances, and comorbid diseases [19].

Chronic inflammation in patients with AD is caused by the impaired epidermal barrier and activation of epidermal pro-inflammatory factors, attracting multiple T cells. T cells are associated with both acute and chronic kidney diseases, as well as renal fibrosis. In addition, several pro-inflammatory components generated from the AD process may impair endothelial function and, consequently, cause kidney vasculature damage or directly lead to kidney damage [17]. As a result, several studies found that AD was the most common dermatological manifestation in chronic kidney disease patients [20,21]. No study has reported the effect of AD and ESRD on infection risk. However, as skin is the source of many systemic infections in ESRD because of the need to frequently access the bloodstream for dialysis, we wished to determine whether a worsening of overall skin integrity due to AD might potentiate the existing risk in ESRD patients. To fill the gap in evidence, the purpose of this study was to determine the increased risk of infection in patients with both ESRD and AD, in a study using data exclusively from the United States Renal Data System (USRDS) [22].

## 2. Materials and Methods

### 2.1. Population

All adult ESRD patients in the USRDS who started dialysis between 2004 and 2019 were eligible for inclusion in the study. Those who were less than 18 years or more than 100 years of age, or had missing or unknown data on age, race, sex, ethnicity, access type, or dialysis type, were excluded. The total sample size was 1,526,266 [22].

### 2.2. Database

The USRDS is a nationally recognized data system that systematically collects, analyzes, and disseminates information pertaining to chronic kidney disease and ESRD within the United States [22]. The USRDS is funded directly by the National Institute of Diabetes and Digestive and Kidney Disease (NIDDK), which increases the credibility and rigor of the data provided. Methodology strictly adheres to established guidelines and best practices in the field of nephrology research. The USRDS staff, in collaboration with key stakeholders such as the Centers for Medicare & Medicaid Services (CMS), the United Network for Organ Sharing (UNOS), and the ESRD networks, work collectively to share datasets and enhance the accuracy of ESRD patient information within the USRDS dataset.

### 2.3. Outcome Variables

Infectious outcomes of interest included bacteremia, septicemia, cellulitis, herpes zoster, and conjunctivitis. Infections following the incident date of dialysis were determined using hospital, detailed, and physician/supplier claims using International Classification of Disease (ICD)-9-CM and ICD-10-CM codes (Supplementary Table S1). The value for the person-years-at-risk was determined as the difference between the first date of the specific infection diagnosis and the incident date of dialysis. For those without an infectious outcome, the person-years-at-risk was determined as the difference between the first date of dialysis and either death or 31 December 2019.

### 2.4. Main Independent Variable—Atopic Dermatitis Diagnosis

Among those included in the sample, a diagnosis of AD, after the incident date of dialysis and prior to the occurrence of the first infectious outcome, was determined from hospital, detailed, and physician/supplier claims using ICD-9-CM and ICD-10-CM codes.

### 2.5. Demographic and Other Clinical Risk Factors

Demographic data including age, race, sex, ethnicity, dialysis modality, and access type were determined from the patient data file or Centers for Medicare & Medicaid Services (CMS) Form 2728. Tobacco use and alcohol dependence were determined from hospital, detailed, or physician/suppler claims using ICD-9-CM and ICD-10-CM codes.

### 2.6. Statistical Analysis

All statistical analysis was performed using SAS 9.4, and statistical significance was assessed using an alpha level of 0.05. Descriptive statistics, including frequencies and percentages or means and standard deviations, where appropriate, on all variables, were determined overall, by AD status, and by each type of infection.

To examine the association of each demographic or clinical risk factor with AD, and to examine the association of AD and demographic and clinical risk factors with each infection, logistic regression was used to conduct a retrospective cohort study. An offset parameter of the natural log of the number of person-years-at-risk was used in the estimation of the relative risk. For AD or for each infection, each risk factor was assessed in a simple, bivariate model. All risk factors were then entered into a full comprehensive logistic regression model for the AD outcome or for each infection outcome, and a backward model-building strategy was used to arrive at the final comprehensive model. Starting with the full model, the least non-significant demographic or clinical risk factor was removed from the model. The Akaike’s information criterion (AIC) and −2Log likelihood (−2LL) test were used to determine whether the reduced model fit was as good as the previous model. A lower AIC and non-statistically significant −2LL test indicated that the reduced model was as good as the previous model. If the reduced model was not as good as the previous model, the variable was re-entered in the model and the next-least non-significant variable was examined for removal. The final model included any demographic or clinical risk factor that was statistically significant and/or needed in the model using the model-building criteria. The adjusted relative risk (aRR) and corresponding 95% confidence interval (CI) are presented for the final models.

## 3. Results

### 3.1. Descriptive Statistics

The USRDS database contained 1,526,270 ESRD patients, enrolled from 2004–2019, who met the inclusion and exclusion criteria. Overall, the mean age was 63.5 years (SD = 14.9), and the majority were of White race (66%) and male sex (57.2%). Nearly all were on hemodialysis (99.9%), and 80.7% had a catheter as their access type. Of these subjects, 2290 (0.2%) had a diagnosis of atopic dermatitis. Table 1 gives the descriptive statistics overall and by AD status. The mean age of AD patients was 62.6 years (SD = 14.7), with the majority being of White race (62.4%) and male (52.1%). All were on hemodialysis (100%), and 75.7% had a catheter as their access type.

### 3.2. Risk Factors Associated with Atopic Dermatitis

As shown in Table 1, in ESRD patients, other race compared with White race, female sex, Hispanic ethnicity, tobacco use, and alcohol dependence were significantly associated with an increased risk of AD. Catheter access compared with AV fistula was associated with a decreased risk of AD.

### 3.3. Associations with Infectious Outcomes of Interest

Supplementary Table S2 shows the descriptive statistics of all variables for bacteremia, septicemia, cellulitis, herpes zoster, and conjunctivitis. Patients with these infectious outcomes were more likely to carry a diagnosis of AD for all infectious outcomes, except conjunctivitis, than those patients without the infection.

As shown in Table 2, after controlling for demographic and clinical covariates, AD was associated with an increased risk of cellulitis and herpes zoster. AD was not associated with an increased risk of bacteremia or conjunctivitis. In simple logistic regression models, AD was associated with an increased risk of septicemia; however, it was confounded by tobacco use, since tobacco use was associated with an increased risk for both AD and septicemia (Supplementary Table S3).

**Figure 1:**
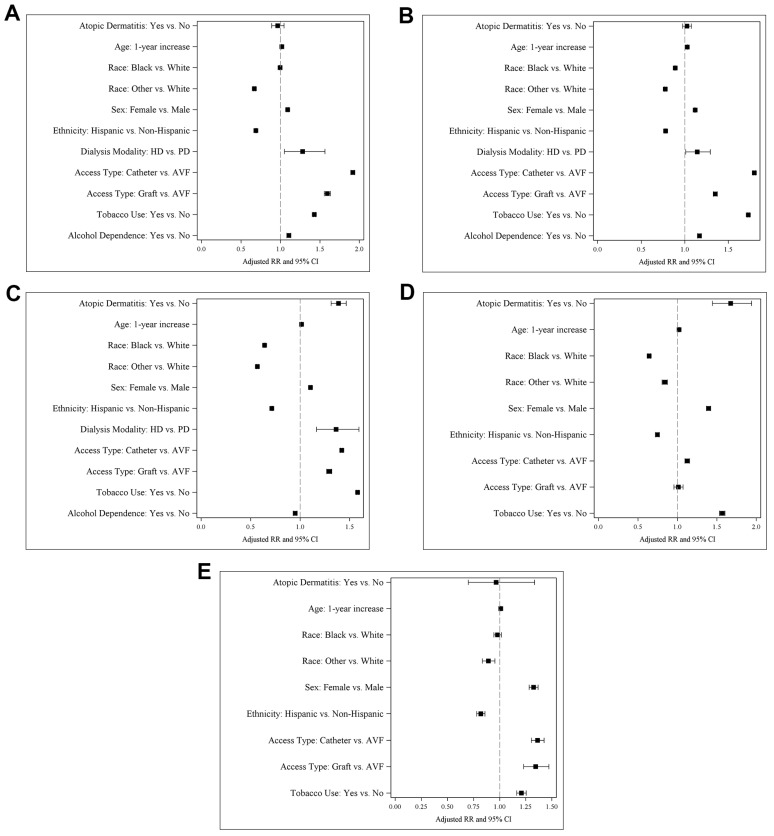
Forest plots for the final multivariable logistic regression models of atopic dermatitis in bacteremia (**A**), septicemia (**B**), cellulitis (**C**), herpes zoster (**D**), and conjunctivitis (**E**).

As shown in Figure 1, in the final models, increasing age was associated with an increased risk of all infections, as was female sex. Hemodialysis compared with peritoneal dialysis was associated with an increased risk of bacteremia, septicemia, and cellulitis, but not herpes zoster or conjunctivitis. Catheter or graft compared with arteriovenous fistula (AVF) was associated with an increased risk of all infections. Tobacco use was also associated with an increased risk of all infections, whereas alcohol dependence was associated with an increased risk of bacteremia and septicemia.

Black race compared with White race was associated with a decreased risk of septicemia, cellulitis, and herpes zoster. Similarly, other race was associated with a decreased risk of all infections, as was Hispanic ethnicity. Alcohol dependence was associated with a decreased risk of cellulitis.

## 4. Discussion

### 4.1. Associations with Infectious Outcomes of Interest

In this study we determined a prevalence of AD in the ESRD population of only 0.2%, much lower than the AD prevalence reported in the general population (approximately 7.3%) [2]. This discrepancy could be due to a focus of the physician treating ESRD patients on clinical management of their kidney failure and other more immediate threats to health rather than dermatological conditions. However, it is most likely attributable to the use of administration codes to identify cases. It should be noted that ICD-9 and -10 codes do not distinguish between childhood-onset AD that is present into adulthood versus adult-onset AD, so these patients may not be exclusively one or the other.

Our results showed that ESRD patients with AD were associated with an increased risk of cellulitis (aRR = 1.39) and herpes zoster (aRR = 1.67), but not bacteremia, septicemia, or conjunctivitis. Patients with ESRD are prone to infections due to the underlying metabolic abnormalities [14]. This study found an associated increase in the risk of cutaneous infections in patients with AD and ESRD, but not mucosal or systemic infections. AD has also been associated with several non-cutaneous infections such as otitis media, pneumonia, and streptococcal throat infection in the general population [23].

Patients with AD are generally chronically colonized by *Staphylococcus aureus* [24]. Scratching at the already poorly functioning epidermal barrier makes it easier for the bacteria to penetrate the skin and leads to infections such as cellulitis (commonly) and bacteremia or septicemia (rarely) [24,25,26]. There are a multitude of case reports depicting the presentation of such systemic infections, even though they are less common [27]. The paucity of systemic infections such as bacteremia and septicemia in patients with AD carries over into the ESRD population. The substantial metabolic derangements in ESRD contribute to higher risk for systemic infections in ESRD in the setting of frequent access to the bloodstream for hemodialysis [28] compared with AD alone. We considered an increase in risk of systemic infections due to worsening of overall skin integrity due to AD; however, our results did not find this association with bacteremia or septicemia. It seems possible that since AD is a disease characterized by an over-activation of the immune system, this hyper-response is protective against bacteria reaching the blood stream.

Similarly, our study found that AD is not associated with an increased risk of conjunctivitis in patients with ESRD. However, in the general population, AD is associated with conjunctivitis and it “is the most common ocular comorbidity in AD”, with a prevalence of 31.7% [29]. In patients with ESRD, a common complaint is irritated, scratchy red eyes [30], which may be difficult to distinguish from conjunctivitis. In addition, patients often have an electrolyte imbalance of calcium and phosphate, which can lead to deposition of the excess in the conjunctiva [31,32]. Although this process is inflammatory, it is not necessarily considered conjunctivitis, which must be ruled out as a diagnosis when patients experience these symptoms [30]. On the other hand, patients with moderate-to-severe AD may also be on immunomodulatory drugs such as dupilumab, which is associated with conjunctivitis [33]. Therefore, we would have expected to see an association with an increased risk of conjunctivitis in patients with AD, if, in fact, they were being treated. However, it is possible that, as we previously observed with psoriasis in the ESRD population [34], patients with AD may also be undertreated.

Additionally, our study found that AD was associated with an increased risk of herpes zoster in ESRD patients. Patients with AD are susceptible to recurrent infections with viruses like herpes zoster [35]. This infection may occur due to stress-induced reactivation from an impaired epidermal skin barrier in AD. Consistent with this idea, ESRD patients with psoriasis also exhibited an increased risk of a diagnosis of herpes zoster, and interestingly, this risk was not mitigated by psoriasis therapy [34]. On the other hand, a systematic review and network meta-analysis demonstrated that treatment with corticosteroids, infliximab, and the JAK inhibitor tofacitinib, as well as several combination therapies, was associated with higher herpes zoster risk in patients with psoriasis and psoriatic arthritis [36]. Similarly, another systematic review and network meta-analysis concluded that the short-term risk of infection (not serious infection) was also higher in patients receiving biologic and small-molecule treatments for psoriasis and psoriatic arthritis [37]. Thus, both treated and untreated patients with psoriasis are likely to be at increased risk for infection. In ESRD patients herpes zoster is known to be associated with increased mortality when combined with other factors such as age and clinical comorbidities [38]. Given the risk of herpes zoster in AD and ESRD independently, it may be helpful in the clinical setting to be aware of these comorbidities and treat complications early. This association also emphasizes the appropriateness of current clinical guidelines recommending vaccinating ESRD patients against herpes zoster.

Of note, a similar study on ESRD patients with psoriasis found an increased risk of all studied infections including bacteremia, septicemia, cellulitis, herpes zoster, and conjunctivitis [34]. Like AD, psoriasis is also an inflammatory condition with a disrupted skin barrier; however, it has a slightly different pathogenesis. Psoriasis is characterized by an overactive Th1 and Th17 immune response compared with the Th2 response primarily seen in AD. Psoriasis often impacts other systems in the body, such as the joints [39]. The increased risk of infections seen in psoriasis could possibly be due to the more systemic nature of the disease compared with AD which seems to be more prone to infections, primarily of the skin.

### 4.2. Demographic and Other Clinical Risk Factors

Other race (aRR = 1.84) and Black race (aRR = 1.05) compared with White race was associated with an increased risk of AD in the ESRD population. In the general population, Black people experience AD at higher rates and with greater severity than White people [40]. However, genetic studies examining polymorphisms that determine AD severity often find that Black people have polymorphisms that are less likely to produce severe AD. Therefore, severity tends to be attributed to a multitude of socioeconomic, environmental, and health factors [40]. It is also unknown whether AD is underdiagnosed in these populations. Since AD can present at varying levels, patients may not seek out health care for the condition, except in severe cases. On the other hand, Black race was protective against all types of infection except bacteremia and conjunctivitis, whereas other race was associated with decreased risk of all types of infection.

Hispanic ethnicity was also associated with an increased risk of AD, although Hispanic ethnicity was found to be protective against all types of infection. Like Black people, those of Hispanic ethnicity also experience greater disease burden than White people, largely due to variations in social determinants of health [40]. It is unknown why these populations may be associated with decreased risk of some types of infection. Perhaps their increased disease burden could lead to better treatment adherence for AD, possibly leading to the lower risk of infection.

Female sex was associated with an increased risk of AD in the ESRD population. In childhood, AD affects males and females equally, but in adulthood, in the general population, it is more prevalent in females [41]. This could be due to females being more likely to seek out medical attention, especially for a cosmetic concern [42]. Being female was also associated with a higher risk of all infections.

Those on hemodialysis compared with peritoneal dialysis were associated with an increased risk of bacteremia, septicemia, and cellulitis. Most included ESRD patients were on hemodialysis (99.9%). Peritoneal dialysis tends to be prescribed for use by patients with better health, fewer comorbidities, of higher socioeconomic classes, and with higher education, likely contributing to its association with a reduced risk of infection [43]. Catheter access and graft (except for herpes zoster) compared with AVF were associated with an increased risk of all types of infection. Patients with catheter access have a higher burden of infection, often related to the site of catheter insertion and the duration of use [44]. Interestingly, catheter access was associated with a decreased risk of AD in the ESRD population. Perhaps patients with AD were considered susceptible to infection; therefore, they were more likely to receive graft or AVF access.

Our research in the ESRD population found that a diagnosis of tobacco use was associated with increased risk of AD. AD is known to be correlated with environmental tobacco smoke in children as well as in the adult onset of the disease [45,46,47]. It is not unexpected that this would be the case in ESRD patients as well. As would be anticipated, tobacco use also increased the risk of all types of infection.

Likewise, our research found that, in ESRD patients, a diagnosis of alcohol dependence was associated with an increased risk of AD. In a study of 100 patients, 25% of those with moderate to severe AD met criteria for an alcohol-use disorder [48]. Alcohol has been found to induce a primarily Th2-focused response in the immune system and to increase IgE levels [49], which may explain the increased risk of AD observed here. Alcohol dependence also increased the risk of bacteremia and septicemia.

### 4.3. Treatments

Patients with AD are treated based on the severity of their symptoms. The initial goal is to prevent flares by maintaining moisture in the skin and avoiding irritating chemicals or clothing. First-line therapy is topical treatments, including corticosteroid creams, ranging in strength from class I to class IV based on their vasoconstricting properties, and calcineurin inhibitors such as pimecrolimus and tacrolimus ointments. For moderate to severe AD, ultraviolet or systemic immunosuppressant therapy may be used as well [50,51]. In patients with AD and ESRD requiring systemic therapy for moderate-to-severe AD, dupilumab is considered the preferred treatment [52]. Cyclosporin has been used for AD and is well tolerated with minimal renal impairment [52,53].

As previously mentioned, patients using treatments such as dupilumab are at an increased risk of conjunctivitis. Some studies have shown the rate of herpes zoster infections is decreased in patients treated with dupilumab and increased in patients treated with Janus kinase inhibitors [54]. Although our study did not investigate whether patients were treated for AD or another disease, the expected impacts were not observed overall. In the case of conjunctivitis, the expected impact would have been an association of AD with an increased rate of conjunctivitis in ESRD patients, which was not observed. Similarly, with treatment we would have expected to see a decrease in the rate of herpes zoster infection; however, in the studied AD group, there was, instead, an association with an increased risk of herpes zoster infection.

### 4.4. Strengths and Limitations

The USRDS is the largest and most representative data system that includes nearly the complete ESRD population in the United States linked to Medicare claims [55]. Using the USRDS dataset ranging from 2004 to 2019, claims, and other information on adult ESRD patients initiating dialysis between 2004 and 2019, we found 2290 of 1,526,266 patients to have a diagnosis of AD (0.2%). Our study showed a much lower prevalence of AD in ESRD patients due to inherent limitations in the USRDS database, including missing coding of AD, underreporting, and misclassification, as well as changes in AD definition and coding over time. These constraints might limit the generalizability of our results, although it is possible that inclusion of patients with AD in the control group instead resulted in an underestimation of the effects of AD in the ESRD population. In addition, the specialty and role of the healthcare professional who included these codes is not able to be inferred through the ICD-9-CM and ICD-10-CM codes. There has been found to be significant overlap in the use of ICD-9-CM codes of 691.8, signifying AD, and 692.9 signifying contact dermatitis [56]. However, our study only looked at ICD-9 code 691.8 for AD. Patients who may have been inaccurately diagnosed with contact dermatitis would not have been included. The prevalence of AD in renal patients is much higher in epidemiological studies, ranging from 2.3% to 4.5% [57]. The concurrence of AD and ESRD is expected to increase due to the common underlying disease development pathway and chronic systemic inflammation, as well as the treatment side effects of AD and environmental and lifestyle factors mentioned above.

## 5. Conclusions

Using the USRDS to examine the relationship between AD and renal disease has not been reported previously. After controlling for demographic and clinical covariates, ESRD patients with AD were determined to be at increased risk for cellulitis and herpes zoster, but not bacteremia, septicemia, or conjunctivitis. Ultimately, AD is an independent risk factor for some infections in ESRD patients. Our findings may add evidence to the link between AD and renal disease, which is needed to better design interventions that may prevent the epidemic rise of the comorbidity of AD and ESRD. In conclusion, reducing the incidence of comorbidity of AD and ESRD will not only reduce associated mortality but also reduce economic and symptom burden, leading to an improved quality of life.

## Figures and Tables

**Table 1:** Descriptive statistics, overall and by atopic dermatitis, and logistic regression results of atopic dermatitis.

					Final Model	
Variable	Level	Overall	Atopic Dermatitis Diagnosis	No Atopic Dermatitis Diagnosis	aRR (95% CI)	*p*-Value
Age		63.5 (14.9)	62.6 (14.7)	63.5 (14.9)		
Race	Black	425,522 (27.9)	643 (28.1)	424,879 (27.9)	1.05 (0.95–1.15)	<0.0001
	Other	93,724 (6.1)	218 (9.5)	93,506 (6.1)	1.84 (1.56–2.12)	
	White	1,007,020 (66.0)	1429 (62.4)	1,005,591 (66.0)		
Sex	Female	652,671 (42.8)	1096 (47.9)	651,575 (42.8)	1.36 (1.25–1.47)	<0.0001
	Male	873,595 (57.2)	1194 (52.1)	872,401 (57.3)		
Ethnicity	Hispanic	230,162 (15.1)	335 (14.6)	229,827 (15.1)	1.19 (1.05–1.34)	0.0059
	Non-Hispanic	1,296,104 (84.9)	1955 (85.4)	1,294,149 (84.9)		
Dialysis	HD	1,525,477 (99.9)	2290 (100.0)	1,523,187 (99.9)		
	PD	789 (0.1)	0 (0.0)	789 (0.1)		
Access type	Catheter	1,232,845 (80.7)	1734 (75.7)	1,231,111 (80.8)	0.75 (0.68–0.83)	<0.0001
	Graft	49,771 (3.3)	102 (4.5)	49,669 (3.3)	1.05 (0.84–1.30)	
	AVF	243,650 (16.0)	454 (19.8)	243,196 (16)		
Tobacco	Yes	273,814 (17.9)	928 (40.5)	272,886 (17.9)	3.24 (2.98–3.53)	<0.0001
	No	1,252,452 (82.1)	1362 (59.5)	1,251,090 (82.1)		
Alcohol dependence	Yes	42,412 (2.8)	114 (5.0)	42,298 (2.8)	1.28 (1.06–1.56)	0.0111
	No	1,483,854 (97.2)	2176 (95.0)	1,481,678 (97.2)		

**Table 2:** Atopic dermatitis as a risk factor for infection.

Infectious Outcome	Atopic Dermatitis vs. No Atopic Dermatitis aRR (95% CI)
Bacteremia	0.96 (0.89–1.05)
Septicemia	1.02 (0.98–1.08)
Cellulitis	1.39 (1.31–1.47)
Herpes zoster	1.67 (1.44–1.94)
Conjunctivitis	0.97 (0.70–1.34)

## Data Availability

The data analyzed in this article were provided by the United States Renal Data System (USRDS) under a data use agreement. The contents of this article do not represent the views of the Department of Veterans Affairs or the United States Government. The data reported here have been supplied by the USRDS. The interpretation and reporting of these data are the responsibility of the authors and in no way should be seen as official policy or an interpretation of the United States Government.

## Supplementary Materials

The following supporting information can be downloaded at: https://www.mdpi.com/article/10.3390/medicina59122145/s1, Table S1: ICD-9-CM and ICD-10-CM codes for outcomes and variables of interest; Table S2: Descriptive statistics by infectious outcomes; Table S3: Logistic regression results of effects of atopic dermatitis on infectious outcomes.

## References

[1] Mohn C.H., Blix H.S., Halvorsen J.A., Nafstad P., Valberg M., Lagerløv P. (2018). Incidence Trends of Atopic Dermatitis in Infancy and Early Childhood in a Nationwide Prescription Registry Study in Norway. JAMA Netw. Open.

[2] Chiesa Fuxench Z.C., Block J.K., Boguniewicz M., Boyle J., Fonacier L., Gelfand J.M., Grayson M.H., Margolis D.J., Mitchell L., Silverberg J.I. (2019). Atopic Dermatitis in America Study: A Cross-Sectional Study Examining the Prevalence and Disease Burden of Atopic Dermatitis in the US Adult Population. J. Investig. Dermatol..

[3] Bylund S., Kobyletzki L.B., Svalstedt M., Svensson Å. (2020). Prevalence and Incidence of Atopic Dermatitis: A Systematic Review. Acta Derm. Venereol..

[4] Arkwright P.D., Koplin J.J. (2023). Impact of a Decade of Research Into Atopic Dermatitis. J. Allergy Clin. Immunol. Pract..

[5] Leung D.Y.M., Boguniewicz M., Howell M.D., Nomura I., Hamid Q.A. (2004). New Insights into Atopic Dermatitis. J. Clin. Investig..

[6] Silverberg J.I., Vakharia P.P., Chopra R., Sacotte R., Patel N., Immaneni S., White T., Kantor R., Hsu D.Y. (2018). Phenotypical Differences of Child- and Adult-Onset Atopic Dermatitis. J. Allergy Clin. Immunol. Pract..

[7] Abuabara K., Margolis D.J. (2013). Do Children Really Outgrow Their Eczema, or Is There More than One Eczema?. J. Allergy Clin. Immunol..

[8] Furue M., Kadono T. (2017). “Inflammatory Skin March” in Atopic Dermatitis and Psoriasis. Inflamm. Res..

[9] Lugović-Mihić L., Meštrović-Štefekov J., Potočnjak I., Cindrić T., Ilić I., Lovrić I., Skalicki L., Bešlić I., Pondeljak N. (2023). Atopic Dermatitis: Disease Features, Therapeutic Options, and a Multidisciplinary Approach. Life.

[10] Guttman-Yassky E., Waldman A., Ahluwalia J., Ong P., Eichenfield L. (2017). Atopic Dermatitis: Pathogenesis. Semin. Cutan. Med. Surg..

[11] Horiuchi Y. (2022). Th1 Regulatory Events by Infectious Pathogens, Herpes Zoster and Herpes Simplex Viruses: Prospects for Therapeutic Options for Atopic Eczema. Adv. Derm. Allergol..

[12] Wang V., Boguniewicz J., Boguniewicz M., Ong P.Y. (2021). The Infectious Complications of Atopic Dermatitis. Ann. Allergy Asthma Immunol..

[13] Hashmi M.F., Benjamin O., Lappin S.L. (2023). End-Stage Renal Disease. StatPearls.

[14] Kato S., Chmielewski M., Honda H., Pecoits-Filho R., Matsuo S., Yuzawa Y., Tranaeus A., Stenvinkel P., Lindholm B. (2008). Aspects of Immune Dysfunction in End-Stage Renal Disease. Clin. J. Am. Soc. Nephrol..

[15] Santoro D., Benedetto F., Mondello P., Pipitò N., Barillà D., Spinelli F., Ricciardi C.A., Cernaro V., Buemi M. (2014). Vascular Access for Hemodialysis: Current Perspectives. Int. J. Nephrol. Renov. Dis..

[16] Lamarche C., Iliuta I.-A., Kitzler T. (2019). Infectious Disease Risk in Dialysis Patients: A Transdisciplinary Approach. Can. J. Kidney Health Dis..

[17] Zhang H., Yuan S., Li Y., Li D., Yu Z., Hu L., Li X., Wang Y., Larsson S.C. (2023). Atopic Dermatitis and Chronic Kidney Disease: A Bidirectional Mendelian Randomization Study. Front. Med..

[18] Schonmann Y., Mansfield K.E., Mulick A., Roberts A., Smeeth L., Langan S.M., Nitsch D. (2021). Inflammatory Skin Diseases and the Risk of Chronic Kidney Disease: Population-Based Case–Control and Cohort Analyses. Br. J. Dermatol..

[19] Galperin T.A., Cronin A.J., Leslie K.S. (2014). Cutaneous Manifestations of ESRD. Clin. J. Am. Soc. Nephrol..

[20] Hay R.J., Johns N.E., Williams H.C., Bolliger I.W., Dellavalle R.P., Margolis D.J., Marks R., Naldi L., Weinstock M.A., Wulf S.K. (2014). The Global Burden of Skin Disease in 2010: An Analysis of the Prevalence and Impact of Skin Conditions. J. Investig. Derm..

[21] Lamb E.J. (2008). United Kingdom Guidelines for Chronic Kidney Disease. Scand. J. Clin. Lab. Investig. Suppl..

[22] Annual Data Report. USRDS. https://adr.usrds.org/.

[23] Langan S.M., Abuabara K., Henrickson S.E., Hoffstad O., Margolis D.J. (2017). Increased Risk of Cutaneous and Systemic Infections in Atopic Dermatitis—A Cohort Study. J. Investig. Derm..

[24] Patel D., Jahnke M.N. (2015). Serious Complications from Staphylococcal Aureus in Atopic Dermatitis. Pediatr. Dermatol..

[25] Hoeger P.H., Ganschow R., Finger G. (2000). Staphylococcal Septicemia in Children with Atopic Dermatitis. Pediatr. Dermatol..

[26] Narla S., Silverberg J.I. (2018). Association between Atopic Dermatitis and Serious Cutaneous, Multiorgan and Systemic Infections in US Adults. Ann. Allergy Asthma Immunol..

[27] Benenson S., Zimhony O., Dahan D., Solomon M., Raveh D., Schlesinger Y., Yinnon A.M. (2005). Atopic Dermatitis—A Risk Factor for Invasive Staphylococcus Aureus Infections: Two Cases and Review. Am. J. Med..

[28] Nguyen D.B., Arduino M.J., Patel P.R. (2019). Hemodialysis-Associated Infections. Chronic Kidney Disease, Dialysis, and Transplantation.

[29] Ravn N.H., Ahmadzay Z.F., Christensen T.A., Larsen H.H.P., Loft N., Rævdal P., Heegaard S., Kolko M., Egeberg A., Silverberg J.I. (2021). Bidirectional Association between Atopic Dermatitis, Conjunctivitis, and Other Ocular Surface Diseases: A Systematic Review and Meta-Analysis. J. Am. Acad. Dermatol..

[30] Mullaem G., Rosner M.H. (2012). Ocular Problems in the Patient with End-Stage Renal Disease. Semin. Dial..

[31] Tokuyama T., Ikeda T., Sato K., Mimura O., Morita A., Tabata T. (2002). Conjunctival and Corneal Calcification and Bone Metabolism in Hemodialysis Patients. Am. J. Kidney Dis..

[32] Alfrey A.C. (2004). The Role of Abnormal Phosphorus Metabolism in the Progression of Chronic Kidney Disease and Metastatic Calcification. Kidney Int..

[33] Blauvelt A., de Bruin-Weller M., Gooderham M., Cather J.C., Weisman J., Pariser D., Simpson E.L., Papp K.A., Hong H.C.-H., Rubel D. (2017). Long-Term Management of Moderate-to-Severe Atopic Dermatitis with Dupilumab and Concomitant Topical Corticosteroids (LIBERTY AD CHRONOS): A 1-Year, Randomised, Double-Blinded, Placebo-Controlled, Phase 3 Trial. Lancet.

[34] Schwade M.J., Tien L., Waller J.L., Davis L.S., Baer S.L., Mohammed A., Young L., Kheda M.F., Bollag W.B. (2021). Treatment of Psoriasis in End-Stage Renal Disease Patients Is Associated with Decreased Mortality: A Retrospective Cohort Study. Am. J. Med. Sci..

[35] Rystedt I., Strannegard I.L., Strannegard O. (1986). Recurrent Viral Infections in Patients with Past or Present Atopic Dermatitis. Br. J. Dermatol..

[36] Chiu H.-Y., Hung Y.-T., Huang S.-W., Huang Y.-H. (2022). Comparative Risk of Herpes Zoster in Patients with Psoriatic Disease on Systemic Treatments: A Systematic Review and Network Meta-Analysis. Ther. Adv. Chronic Dis..

[37] Chiu H.-Y., Hung Y.-T., Huang Y.-H. (2023). Comparative Short-Term Risks of Infection and Serious Infection in Patients Receiving Biologic and Small-Molecule Therapies for Psoriasis and Psoriatic Arthritis: A Systemic Review and Network Meta-Analysis of Randomized Controlled Trials. Ther. Adv. Chronic Dis..

[38] Ahn J.H., Waller J.L., Baer S.L., Colombo R.E., Kheda M.F., Nahman N.S., Turrentine J.E. (2019). Mortality Risk after Herpes Zoster Infection in End-Stage Renal Disease Patients. Clin. Kidney J..

[39] Rendon A., Schäkel K. (2019). Psoriasis Pathogenesis and Treatment. Int. J. Mol. Sci..

[40] Croce E.A., Levy M.L., Adamson A.S., Matsui E.C. (2021). Reframing Racial and Ethnic Disparities in Atopic Dermatitis in Black and Latinx Populations. J. Allergy Clin. Immunol..

[41] Johansson E.K., Bergström A., Kull I., Melén E., Jonsson M., Lundin S., Wahlgren C.-F., Ballardini N. (2022). Prevalence and Characteristics of Atopic Dermatitis among Young Adult Females and Males—Report from the Swedish Population-Based Study BAMSE. J. Eur. Acad. Dermatol. Venereol..

[42] Bannister M.J., Freeman S. (2000). Adult-Onset Atopic Dermatitis. Australas. J. Dermatol..

[43] Sinnakirouchenan R., Holley J.L. (2011). Peritoneal Dialysis Versus Hemodialysis: Risks, Benefits, and Access Issues. Adv. Chronic Kidney Dis..

[44] Miller L.M., Clark E., Dipchand C., Hiremath S., Kappel J., Kiaii M., Lok C., Luscombe R., Moist L., Oliver M. (2016). Hemodialysis Tunneled Catheter-Related Infections. Can. J. Kidney Health Dis..

[45] Yi O., Kwon H.-J., Kim H., Ha M., Hong S.-J., Hong Y.-C., Leem J.-H., Sakong J., Lee C.G., Kim S.-Y. (2012). Effect of Environmental Tobacco Smoke on Atopic Dermatitis among Children in Korea. Environ. Res..

[46] Lee C.H., Chuang H.Y., Hong C.H., Huang S.K., Chang Y.C., Ko Y.C., Yu H.S. (2011). Lifetime Exposure to Cigarette Smoking and the Development of Adult-onset Atopic Dermatitis. Br. J. Dermatol..

[47] Abdualrasool M., Al-Shanfari S., Booalayan H., Boujarwa A., Al-Mukaimi A., Alkandery O., Akhtar S. (2018). Exposure to Environmental Tobacco Smoke and Prevalence of Atopic Dermatitis among Adolescents in Kuwait. Dermatology.

[48] Gilhooley E., O’Grady C., Roche D., Mahon J.M., Hambly R., Kelly A., Dhonncha E.N., Moriarty B., Connolly M., Kirby B. (2021). High Levels of Psychological Distress, Sleep Disturbance, and Alcohol Use Disorder in Adults With Atopic Dermatitis. Dermatitis.

[49] Linneberg A., Gonzalez-Quintela A. (2016). The Unsolved Relationship of Alcohol and Asthma. Int. Arch. Allergy Immunol..

[50] Thomsen S.F. (2014). Atopic Dermatitis: Natural History, Diagnosis, and Treatment. ISRN Allergy.

[51] Frazier W., Bhardwaj N. (2020). Atopic Dermatitis: Diagnosis and Treatment. Am. Fam. Physician.

[52] Drucker A.M., Lam M., Flohr C., Thyssen J.P., Kabashima K., Bissonnette R., Dlova N.C., Aoki V., Chen M., Yu J. (2022). Systemic Therapy for Atopic Dermatitis in Older Adults and Adults With Comorbidities: A Scoping Review and International Eczema Council Survey. Dermatitis.

[53] Choi E., Cook A., Phuan C., Martin A., Yang S., Aw D., Chandran N.S. (2021). Outcomes of Prolonged and Low-Dose Ciclosporin in an Asian Population. J. Dermatol. Treat..

[54] Adam D.N., Gooderham M.J., Beecker J.R., Hong C.H., Jack C.S., Jain V., Lansang P., Lynde C.W., Papp K.A., Prajapati V.H. (2023). Expert Consensus on the Systemic Treatment of Atopic Dermatitis in Special Populations. J. Eur. Acad. Dermatol. Venereol..

[55] Foley R.N., Collins A.J. (2013). The USRDS: What You Need to Know about What It Can and Can’t Tell Us about ESRD. Clin. J. Am. Soc. Nephrol..

[56] Hsu D.Y., Dalal P., Sable K.A., Voruganti N., Nardone B., West D.P., Silverberg J.I. (2017). Validation of International Classification of Disease Ninth Revision Codes for Atopic Dermatitis. Allergy.

[57] Egeberg A., Andersen Y.M.F., Gislason G.H., Skov L., Thyssen J.P. (2017). Prevalence of Comorbidity and Associated Risk Factors in Adults with Atopic Dermatitis. Allergy.

