# Supplementary material for: Atopic Dermatitis and the Risk of Infection in End-Stage Renal Disease
Source: Medicina (Kaunas). 2023 Dec 10;59(12):2145. doi: 10.3390/medicina59122145 (PMC10744789; doi:10.3390/medicina59122145)
Supplement: Supplementary file 1 [file medicina-59-02145-s001.zip › medicina-2707898-SI.pdf]

Supplementary Table S1: ICD-9-CM and ICD-10-CM Codes for Outcomes and Variables of Interest		
	ICD-9-CM	ICD-10-CM
Atopic Dermatitis	691.8	L20.0, L20.81, L20.82, L20.84, L20.89, L20.9
Bacteremia	790.7	R78.81
Septicemia	038.0, 038.1, 038.11, 038.2, 038.3, 038.4, 038.8, 038.9	A40.3, A40.9, A41.01, A41.89, A41.9, A41.4
Conjunctivitis	372.3, 372.30, 077.3, 372.03	H10.0, H10.01, H10.011, H10.012, H10.013, H10.019, H10.02, H10.021, H10.022, H10.023, H10.029, H10.23, H10.231, H10.23, H10.233, H10.239, H10.3, H10.30, H10.31, H10.32, H10.33, H10.33, H10.89, H10.9
Cellulitis	682.0, 682.1, 682.2, 682.3, 682.4, 682.5, 682.6, 682.7, 682.8, 682.9,	L030.01, L030.011, L030.012, L030.019, L03.03, L030.031, L030.032, L030.039, L03.1, L03.11, L03.111, L03.112, L03.113, L03.114, L03.115, L03.116, L03.119, L03.2, L03.211, L03.213, L03.22, L03.221, L03.3, L03.31, L03.311, L03.312, L03.313, L03.314, L03.315, L03.316, L03.317, L03.319, L03.9, L03.90, L03.8, L03.81, L03.811, L03.818
Herpes Zoster	053.0, 053.1, 053.10, 053.11, 053.12, 053.13, 053.14, 053.19, 053.2, 053.20, 053.21, 053.22, 053.29, 053.7, 053.71, 053.79, 053.8, 053.9	B02.0, B02.1, B02.2, B02.21, B02.22, B02.23, B02.24, B02.29, B02.3, B02.30, B02.31, B02.32, B02.33, B02.34, B02.39, B02.7, B02.8, B02.9
Tobacco Use	305.1, V15.82	Z87.891, Z72.0, F17.220
Alcohol Dependence	305.0, 305.01, 305.02, 305.03, 303.9, 303.91, 303.92, 303.93	F10.98, F10.99, F10.1, F10.11, F10.90, F10.20, F10.21

Supplementary Table S2: Descriptive Statistics by Infectious Outcomes

		Bacteremia		Septicemia		Cellulitis		Herpes Zoster		Conjunctivitis	
Variable	Level	Yes N=236581 (15.5%)	No N=1289685 (84.5%)	Yes N=549363 (36.0%)	No N=976903 (64.0%)	Yes N=363024 (23.8%)	No N=1163243 (76.2%)	Yes N=39892 (2.6%)	No N=1486374 (97.4%)	Yes N=15405 (1.0%)	No N=151086 1 (99.0%)
**Main Independent Variable**											
Atopic Dermatitis	Yes	593 (0.3)	1697 (0.1)	1573 (0.3)	717 (0.1)	1256 (0.4)	1034 (0.1)	175 (0.4)	2115 (0.1)	37 (0.2)	2253 (0.2)
	No	235988 (99.8)	1287988 (99.9)	547790 (99.7)	976186 (99.9)	361768 (99.7)	1162208 (99.9)	39717 (99.6)	1484259 (99.9)	15368 (99.8)	1508608 (99.9)
**Demographic and Clinical Risk Factors**											
Age (years) – mean (SD)		62.3 (14.8)	63.7 (14.8)	64.5 (14.3)	63 (15.1)	62.5 (14.3)	63.8 (15.0)	63.6 (14.5)	63.5 (14.9)	61.2 (16.1)	63.5 (14.8)
Race – n (%)	Black	78047 (33.0)	347475 (26.9)	159633 (29.1)	265889 (27.2)	91859 (25.3)	333663 (28.7)	9531 (23.9)	415991 (28.0)	5032 (32.6)	420490 (27.8)
	Other	11648 (4.9)	82076 (6.4)	30738 (5.6)	62986 (6.5)	17575 (4.8)	76149 (6.6)	2661 (6.7)	91063 (6.1)	979 (6.4)	92745 (6.2)
	White	146886 (62.1)	860134 (66.7)	358992 (65.4)	648028 (66.3)	253590 (69.9)	753430 (64.8)	27700 (69.4)	979320 (65.9)	9394 (61.0)	997626 (66.0)
Sex – n (%)	Female	106191 (44.9)	546480 (42.4)	247470 (45.1)	405201 (41.5)	160395 (44.2)	492276 (42.3)	20027 (50.2)	632644 (42.6)	7676 (49.8)	644995 (42.7)
	Male	130390 (55.1)	743205 (57.6)	301893 (55)	571702 (58.5)	202629 (55.8)	670966 (57.7)	19865 (49.8)	853730 (57.4)	7729 (50.2)	865866 (57.3)
Ethnicity – n (%)	Hispanic	29515 (12.5)	200647 (15.6)	73919 (13.5)	156243 (16.0)	51710 (14.2)	178452 (15.3)	5589 (14.0)	224573 (15.1)	2219 (14.4)	227943 (15.1)
	Non-Hispanic	207066 (87.5)	1089038 (84.4)	475444 (86.5)	820660 (84.0)	311314 (85.8)	984790 (84.7)	34303 (86.0)	1261801 (84.9)	13186 (85.6)	1282918 (84.9)
Dialysis Modality – n (%)	HD	236482 (99.9)	1288995 (99.9)	549113 (99.9)	976364 (99.9)	362868 (99.9)	1162609 (99.9)	39867 (99.9)	1485610 (99.9)	NR	1510078 (99.9)
	PD	99 (0.1)	690 (0.1)	250 (0.1)	539 (0.1)	156 (0.1)	633 (0.1)	25 (0.1)	764 (0.1)	NR	783 (0.1)
Access Type – n (%)	Catheter	199842 (84.5)	1033003 (80.1)	458251 (83.4)	774594 (79.3)	294498 (81.1)	938347 (80.7)	31285 (78.5)	1201560 (80.8)	12488 (81.1)	1220357 (80.8)
	Graft	8482 (3.6)	41289 (3.2)	18313 (3.3)	31458 (3.2)	12724 (3.5)	37047 (3.2)	1409 (3.5)	48362 (3.3)	621 (4.0)	49150 (3.3)
	AVF	28257 (11.9)	215393 (16.7)	72799 (13.3)	170851 (17.5)	55802 (15.4)	187848 (16.2)	7198 (18.0)	236452 (15.9)	2296 (14.9)	241354 (16.0)
Tobacco – n (%)	Yes	64343 (27.2)	209471 (16.2)	162087 (29.5)	111727 (11.4)	102901 (28.4)	170913 (14.7)	11264 (28.2)	262550 (17.7)	3643 (23.7)	270171 (17.9)
	No	172238 (72.8)	1080214 (83.8)	387276 (70.5)	865176 (88.6)	260123 (71.7)	992329 (85.3)	28628 (71.8)	1223824 (82.3)	11762 (76.4)	1240690 (82.1)
Alcohol Dependence – n (%)	Yes	8524 (3.6)	33888 (2.6)	20397 (3.7)	22015 (2.3)	11971 (3.3)	30441 (2.6)	1192 (3.0)	41220 (2.8)	470 (3.0)	41942 (2.8)
	No	228057 (96.4)	1255797 (97.4)	528966 (96.3)	954888 (97.8)	351053 (96.7)	1132801 (97.4)	38700 (97.0)	1445154 (97.2)	14935 (97.0)	1468919 (97.2)

Supplementary Table S3: Logistic Regression Results of Atopic Dermatitis on Infectious Outcomes											
Variable	Level	Simple Models: RR (95% CI) p-value					Final Models: aRR (95% CI) p-value				
		Bacteremia	Septicemia	Cellulitis	Herpes Zoster	Conjunctivitis	Bacteremia	Septicemia	Cellulitis	Herpes Zoster	Conjunctivitis
Main Independent Variable											
Atopic Dermatitis	Yes vs. No	1.05 (0.97-1.13) 0.2861	1.21 (1.15-1.26) <0.0001	1.57 (1.49-1.65) <0.0001	1.90 (1.64-2.20) <0.0001	1.02 (0.74-1.41) 0.9108	0.96 (0.89-1.05) 0.3743	1.02 (0.98-1.08) 0.3357	1.39 (1.31-1.47) <.0001	1.67 (1.44-1.94) <.0001	0.97 (0.70-1.34) 0.8338
Demographic and Clinical Risk Factors											
Age (years)	1year increase	1.016 (1.016-1.016) <0.0001	1.027 (1.027-1.028) <0.0001	1.017 (1.017-1.017) <0.0001	1.023 (1.023-1.024) <0.0001	1.011 (1.010-1.013) <0.0001	1.016 (1.016-1.016) <.0001	1.027 (1.027-1.028) <.0001	1.014 (1.014-1.015) <.0001	1.021 (1.02-1.021) <.0001	1.011 (1.009-1.012) <.0001
Race	Black vs. White	1.03 (1.02-1.04) <0.0001	0.85 (0.85-0.86) <0.0001	0.66 (0.66-0.67) <0.0001	0.64 (0.62-0.65) <0.0001	1.00 (0.97-1.04) 0.8867	1.00 (0.99-1.01) 0.3622	0.89 (0.88-0.90) <.0001	0.64 (0.64-0.65) <.0001	0.64 (0.63-0.66) <.0001	0.98 (0.94-1.02) 0.2535
	Other vs. White	0.68 (0.66-0.69) <0.0001	0.73 (0.72-0.74) <0.0001	0.57 (0.56-0.58) <0.0001	0.83 (0.80-0.87) <0.0001	0.91 (0.85-0.97) 0.0038	0.67 (0.66-0.68) <.0001	0.78 (0.77-0.79) <.0001	0.57 (0.56-0.58) <.0001	0.84 (0.81-0.87) <.0001	0.89 (0.84-0.95) 0.0009
Sex	Female vs. Male	1.12 (1.11-1.13) <0.0001	1.14 (1.13-1.15) <0.0001	1.09 (1.08-1.10) <0.0001	1.38 (1.34-1.40) <0.0001	1.35 (1.31-1.39) <0.0001	1.09 (1.08-1.10) <.0001	1.12 (1.11-1.13) <.0001	1.11 (1.10-1.11) <.0001	1.39 (1.37-1.42) <.0001	1.32 (1.28-1.37) <.0001
Ethnicity	Hispanic vs. Non-Hispanic	0.65 (0.65-0.66) <0.0001	0.71 (0.71-0.72) <0.0001	0.77 (0.76-0.78) <0.0001	0.76 (0.74-0.78) <0.0001	0.79 (0.75-0.83) <0.0001	0.69 (0.68-0.70) <.0001	0.78 (0.77-0.79) <.0001	0.71 (0.71-0.72) <.0001	0.75 (0.72-0.77) <.0001	0.82 (0.78-0.86) <.0001
Dialysis Modality	HD vs. PD	1.37 (1.13-1.67) 0.0016	1.28 (1.13-1.45) 0.0001	1.39 (1.19-1.63) <0.0001	0.89 (0.61-1.32) 0.5724	1.44 (0.65-3.21) 0.3738	1.28 (1.05-1.56) 0.0150	1.14 (1.01-1.29) 0.0330	1.36 (1.17-1.59) 0.0001	[redacted]	[redacted]
Access Type	Catheter vs. AVF	1.85 (1.82-1.87) <0.0001	1.70 (1.68-1.71) <0.0001	1.35 (1.33-1.36) <0.0001	1.08 (1.05-1.11) <0.0001	1.35 (1.29-1.41) <0.0001	1.92 (1.89-1.94) <.0001	1.80 (1.78-1.81) <.0001	1.42 (1.41-1.43) <.0001	1.12 (1.10-1.15) <.0001	1.36 (1.30-1.43) <.0001
	Graft vs. AVF	1.69 (1.64-1.73) <0.0001	1.43 (1.41-1.46) <0.0001	1.27 (1.25-1.30) <0.0001	1.05 (0.99-1.11) 0.0814	1.46 (1.33-1.59) <0.0001	1.59 (1.55-1.63) <.0001	1.35 (1.33-1.37) <.0001	1.29 (1.27-1.32) <.0001	1.01 (0.96-1.07) 0.6845	1.35 (1.23-1.47) <.0001
Tobacco	Yes vs. No	1.46 (1.45-1.48) <0.0001	1.73 (1.72-1.74) <0.0001	1.60 (1.59-1.61) <0.0001	1.51 (1.48-1.55) <0.0001	1.19 (1.14-1.23) <0.0001	1.43 (1.42-1.44) <.0001	1.73 (1.72-1.74) <.0001	1.58 (1.57-1.59) <.0001	1.57 (1.53-1.60) <.0001	1.21 (1.16-1.26) <.0001
Alcohol Dependence	Yes vs. No	1.22 (1.19-1.24) <0.000	1.28 (1.26-1.29) <0.0001	1.11 (1.09-1.13) <0.0001	0.99 (0.93-1.05) 0.6596	1.01 (0.92-1.11) 0.8528	1.11 (1.08-1.13) <.0001	1.17 (1.15-1.19) <.0001	0.95 (0.93-0.97) <.0001	[redacted]	[redacted]
